# The disease progression of end-stage atrial cardiomyopathy over three decades: a case report

**DOI:** 10.1093/ehjcr/ytae530

**Published:** 2024-10-10

**Authors:** Takafumi Oka, Takayuki Sekihara, Kentaro Ozu, Tomoaki Nakano, Yasushi Sakata

**Affiliations:** Department of Cardiovascular Medicine, Osaka University Graduate School of Medicine, 2-2 Yamadaoka, Suita, Osaka 565-0871, Japan; Department of Cardiovascular Medicine, Osaka University Graduate School of Medicine, 2-2 Yamadaoka, Suita, Osaka 565-0871, Japan; Department of Cardiovascular Medicine, Osaka University Graduate School of Medicine, 2-2 Yamadaoka, Suita, Osaka 565-0871, Japan; Department of Cardiovascular Medicine, Osaka University Graduate School of Medicine, 2-2 Yamadaoka, Suita, Osaka 565-0871, Japan; Department of Cardiovascular Medicine, Osaka University Graduate School of Medicine, 2-2 Yamadaoka, Suita, Osaka 565-0871, Japan

**Keywords:** Atrial cardiomyopathy, Cerebral infarction, Left atrial thrombus, Congestive heart failure, Cardiac resynchronization therapy, Case report

## Abstract

**Background:**

Atrial cardiomyopathy (AtCM) has drawn attention as the pathophysiology related to cardiovascular events such as atrial tachyarrhythmia, congestive heart failure, and embolic stroke. As the concept of AtCM is relatively recent, the long-term clinical course of AtCM has not been reported.

**Case summary:**

Here, we describe a 78-year-old patient diagnosed with end-stage AtCM. He had started to visit our hospital due to paroxysmal atrial fibrillation (AF) and hypertrophic cardiomyopathy over three decades since the age of 45. During follow-up, he experienced cardiogenic embolism and pacemaker implantation due to sick sinus syndrome. At this time, he complained of palpitation due to AF and underwent catheter ablation. Regardless of *de novo* ablation, left atrial voltage mapping showed ultimately extensive scar in left atrium and pulmonary vein, suggesting that conventional AF ablation strategy was ineffective. From this finding, he was diagnosed with end-stage AtCM. In the review of the previous 12-lead electrocardiogram, P-wave amplitude was decreased, and PR duration was prolonged gradually. We performed only cavotricuspid isthmus ablation and ended the ablation session. After six months, he complained of dyspnoea on effort due to pacing-induced cardiomyopathy. Furthermore, before the cardiac resynchronization therapy with a defibrillator (CRT-D) upgrade, left atrial appendage thrombus was detected even under the administration of apixaban. After thrombolysis with warfarin, CRT-D upgrade the left ventricular ejection fraction was improved.

**Discussion:**

In this case, the patient slowly developed end-stage AtCM and experienced multiple cardiovascular events related to severe AtCM. We should care for the disease progression of AtCM with vigilance.

**Learning points**

- To diagnose patients with atrial cardiomyopathy (AtCM), medical history and the findings of multiple modalities, such as 12-lead electrocardiogram, transoesophageal echocardiogram, cardiac computed tomography, magnetic resonance imaging, and electrophysiological voltage mapping, should be considered.
- Patients with AtCM should be carefully followed up to monitor arrhythmia, embolic stroke, and congestive heart failure.
- For patients with AtCM, an optimal treatment option should be selected based on clinical manifestations during the long-term course.

## Introduction

Atrial cardiomyopathy (AtCM) has drawn attention as the pathophysiology related to cardiovascular events such as atrial tachyarrhythmia, congestive heart failure (CHF), and embolic stroke.^1–3^ Since the concept of AtCM is relatively recent, the long-term clinical course has not been reported. Herein, we present a case with end-stage AtCM progressing over three decades. From his clinical manifestations, we learned about the treatment of patients with end-stage AtCM.

## Summary figure

**Figure:**
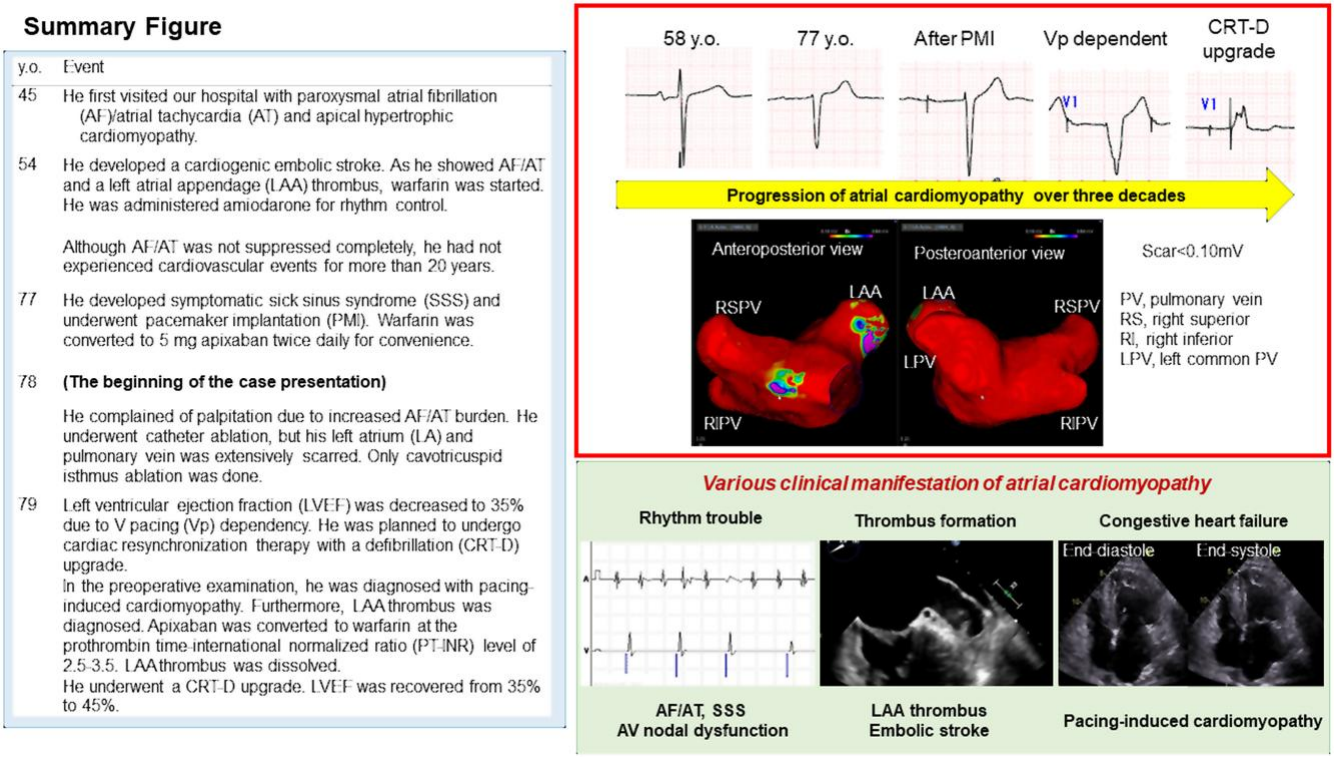


## Case presentation

**Figure 1:**
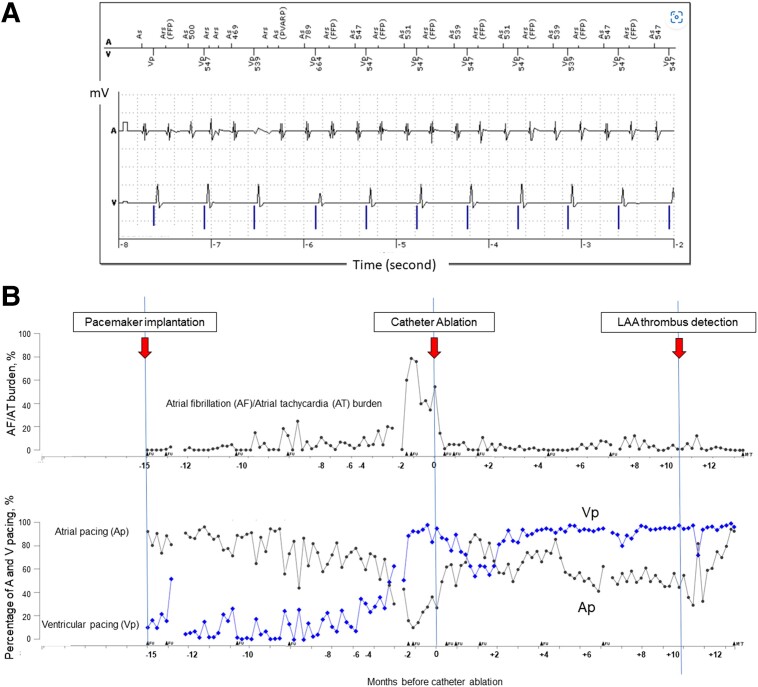
(*A*) The electrocardiogram of atrial tachyarrhythmia stored in the pacemaker. (*B*) AF/AT burden (upper) and %Ap/%Vp (lower). The date of catheter ablation is set as day 0 in all figures.

**Figure 2:**
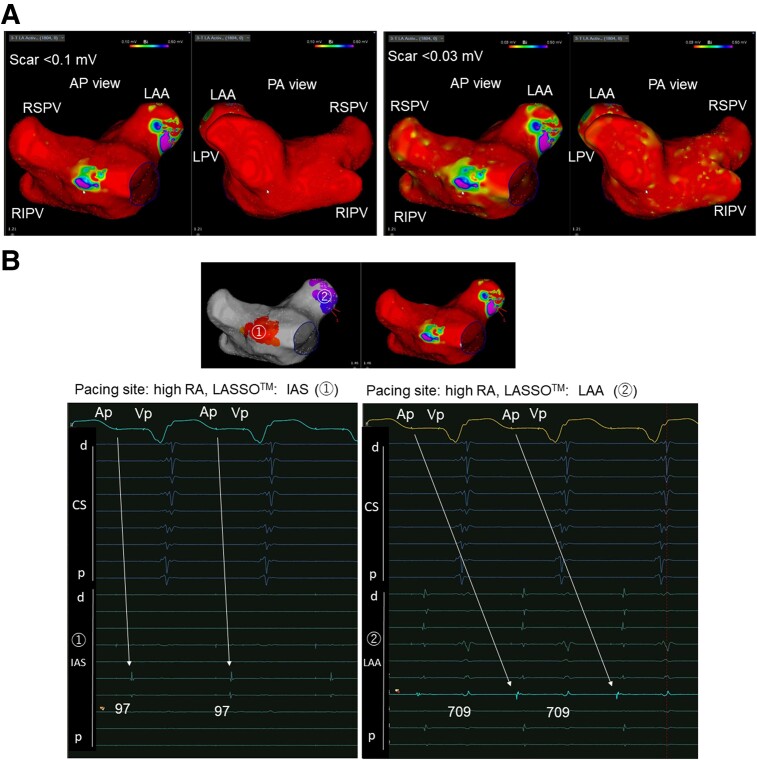
(*A*) Voltage mapping of the left atrium (LA) and pulmonary veins (PVs) under right atrial (RA) pacing. Scar was defined as <0.10 mV (left) and <0.03 mV (right). RS, right superior; RI, right inferior; LPV, left common PV; LAA, LA appendage. (*B*) Local electrical delay from RA pacing in intra-atrial septum (IAS) (left) and LAA (right).

**Figure 3:**
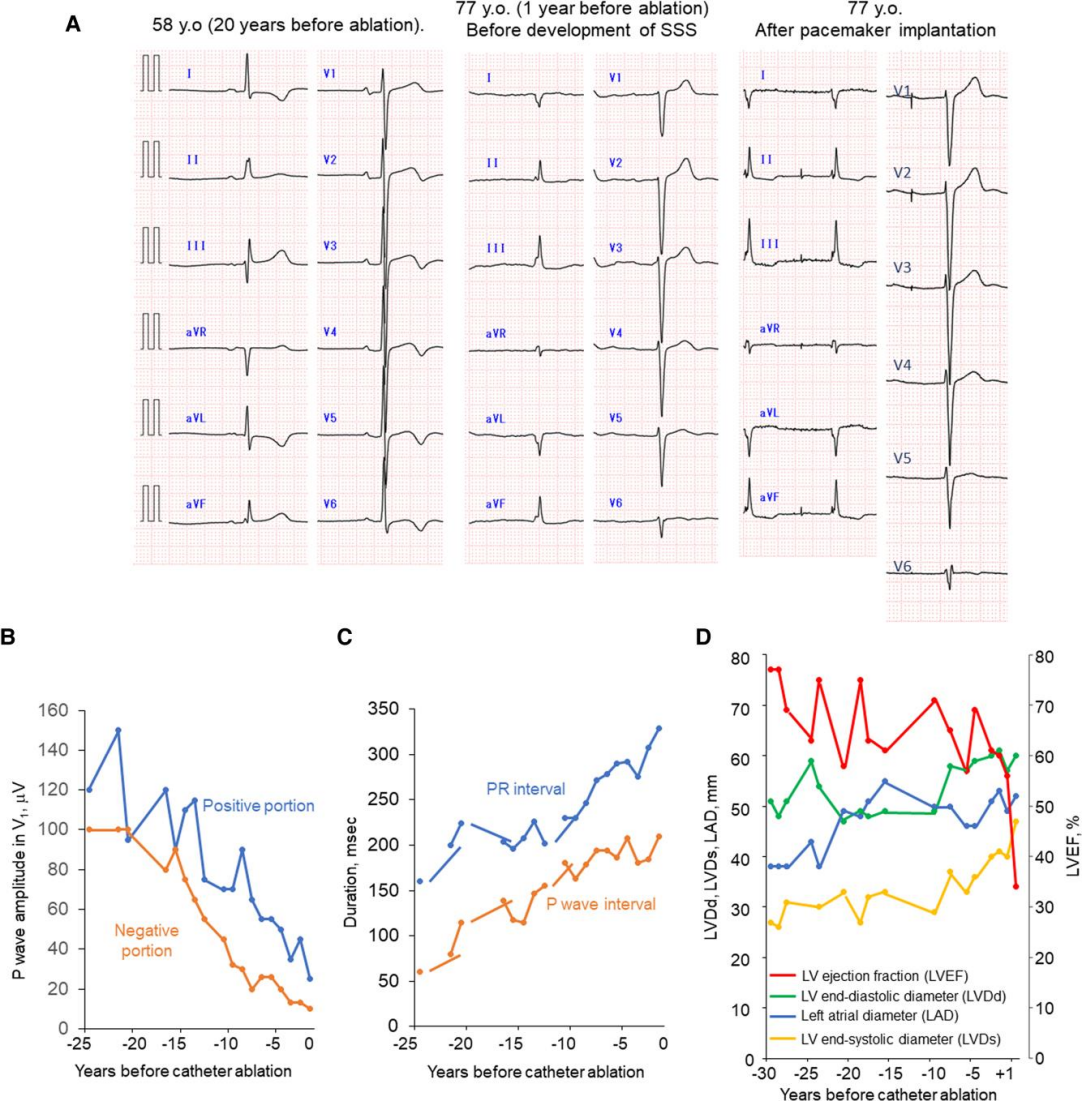
(*A*) Series of 12-lead ECG. (*B*) Time course of the P-wave amplitude in V_1_ lead. (*C*) PR interval and P-wave duration. (*D*) Echocardiographic parameters.

A 78-year-old male (weight: 81 kg, height 1.69 m, CHA_2_DS_2_-VASc score: 6) with a history of paroxysmal atrial fibrillation (AF)/atrial tachycardia (AT), embolic stroke, apical hypertrophic cardiomyopathy (HCM), and pacemaker implantation due to sick sinus syndrome presented with worsening palpitations due to AF/AT (*Figure 1A*). Regardless of symptom, he had refused to undergo catheter ablation before. The AF/AT burden increased as the ventricular pacing (Vp) burden increased (*Figure 1B*). We recommended catheter ablation. On admission, he showed no sign of CHF. He was prescribed with 0.625 mg of bisoprolol, 25 mg of eplerenone, 8 mg of candesartan, 100 mg of amiodarone daily, and 5 mg of apixaban twice daily. The left ventricular ejection fraction (LVEF) was 56%, the left atrial (LA) diameter was 49 mm. Cardiac computed tomography (CCT) showed no signs of coronary artery disease. The transoesophageal echocardiogram did not show LA thrombus. In the ablation session, the pulmonary vein (PV) potential was not recorded with a 20-pole ring catheter (LASSO™, Biosense Webstar, Diamond Bar, CA, USA). The voltage mapping under right atrial pacing exhibited extreme scarring of the LA and PVs (*Figure 2A*). The LA potential was almost invisible, except for part of the interatrial septum, and a delayed left atrial appendage (LAA) potential was observed behind the paced QRS (*Figure 2B*). Based on the voltage mapping findings, he was diagnosed with end-stage AtCM. Pulmonary vein isolation and LA ablation seemed ineffective. Spontaneous unmappable AF/AT occurred. As the AT sequence transiently resembled a common atrial flutter, we created a cavotricuspid isthmus (CTI) block line and ended the session. After the session, the AF/AT burden decreased (*Figure 1B*). In the review of the 12-lead electrocardiogram (ECG, *Figure 3A*), the amplitude of the negative portion of the P-wave in V_1_ was reduced (*Figure 3B*), and the PR interval was prolonged (*Figure 3C*), suggesting a progression of atrial scarring. LA dilatation had gradually progressed (*Figure 3D*). The LA volume index was 91.7 mL/m^2^, and the total LA emptying fraction measured with CCT was reduced to 1.1%.

**Figure 4:**
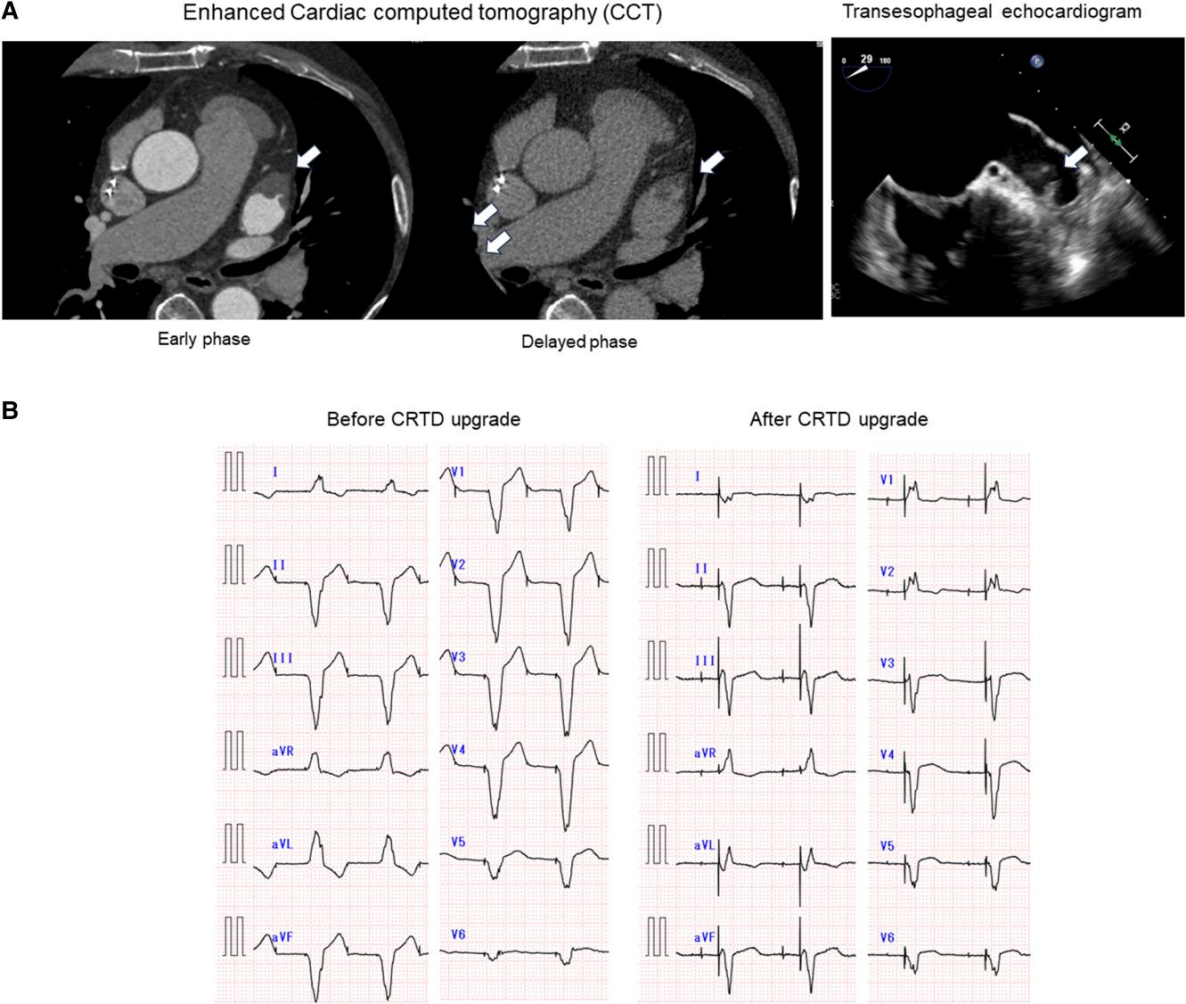
(*A*) LAA thrombus detected with enhanced cardiac CT and transoesophageal echocardiogram. (*B*) Twelve-lead ECG before and after CRT-D upgrade.

Six months after ablation, he complained of dyspnoea on exertion. He became Vp-dependent (*Figure 1B*), and LVEF decreased to 35% with dyssynchrony under 0.625 mg of bisoprolol, 25 mg of eplerenone, and 8 mg of candesartan daily. The level of N-terminal pro B-type natriuretic peptide (NT-proBNP) increased from 400 to 800 pg/mL. Right ventricular biopsy showed no specific changes indicative of end-stage HCM and cardiac amyloidosis, ruling out these diseases. Furthermore, whole exome sequencing revealed no pathogenic variants of *MYL4* or *NPPA*, both of which are known genetic causes of AtCM. We concluded that he had developed pacing-induced cardiomyopathy. As non-sustained VT was detected, we planned a cardiac resynchronization therapy with a defibrillator (CRT-D) upgrade. However, pre-operative examination incidentally revealed an LAA thrombus (*Figure 4A*), although he had regularly taken 5 mg of apixaban twice daily and the recent AF/AT burden was <10%. He had no comorbidity that enhanced coagulability. Apixaban was converted to warfarin at the prothrombin time-international normalized ratio (PT-INR) level of 2.5–3.0, and the thrombus disappeared within two months. After the CRT-D upgrade and conversion from candesartan to 50 mg of sacubitril valsartan, CHF symptom was improved (*Figure 4B*) and LVEF recovered to 45%.

## Discussion

Here, we describe a case of end-stage AtCM. Over three decades, he experienced various AtCM-related cardiovascular events. To the best of our knowledge, this is the first report of such a long-term clinical course of end-stage AtCM.

Recently, AtCM has drawn attention as the pathophysiology related to cardiovascular events.^1–3^ In the classification of different stages of AtCM,^4^ the patient was diagnosed with severe AtCM because of atrial systolic failure, major degrees of interstitial alteration, and severe atrial enlargement. We diagnosed him with ‘end-stage’ AtCM from the extremely extensive scar of the voltage mapping. As non-invasive modalities, speckle-tracking echocardiography and magnetic resonance imaging (MRI) are generally performed for the diagnosis and staging. Since artefact of pacemaker could disturb the examination, we did not perform MRI with late-gadolinium enhancement. His AtCM might have been triggered by multiple aetiological factors such as HCM, AF/AT, and hypertension. Patients with HCM frequently have atrial fibrosis due to haemodynamic and genetic factors.^5^ Furthermore, AF/AT is the cause and effect of AtCM.^1,3^ Since voltage mapping showed extensive scar, the substrate modification, such as linear ablation, scar homogenization, and scar isolation^2^ seemed ineffective. However, CTI block was partially effective, suggesting that common atrial flutter could contribute to the mechanism of AF/AT. To achieve further frequency control, atrioventricular nodal ablation plus CRT might be considered. Retrospectively, an early rhythm control strategy by ablation might have slowed down the disease progression.^6,7^

The patient experienced LAA thrombus under direct oral anti-coagulation (DOAC) administration and a small AF burden. Since the first symptomatic cardiogenic embolism was identified two decades ago, thromboembolism had been prevented with warfarin. He had no comorbidity enhancing coagulability. Progression of LAA scarring, increasing LA pressure due to CHF worsening, and conversion from warfarin to a DOAC might cause recurrent thrombus. Atrial cardiomyopathy increases thrombogenicity because of endothelial dysfunction, hypercoagulability, and LAA systolic dysfunction.^8^ The combination of warfarin at high PT-INR control with LAA closure might increase the effectiveness for secondary prevention.^9^ The optimal anti-coagulation strategy in patients with end-stage AtCM should be investigated in the future.

Atrial cardiomyopathy causes LA dysfunction and AF/AT, both of which contribute to CHF worsening. PR prolongation resulted in Vp-dependency, which increased AF/AT burden^10^ and provoked pacing-induced cardiomyopathy.^11^ PR prolongation is caused by atrial remodelling that slows conduction velocity and impairs the compact atrioventricular node, a part of the atrium not insulated from the atrial working myocytes.^12,13^

Altogether, patients with end-stage AtCM are at high risk of multiple cardiovascular events. Temporal evaluation of AtCM staging should be performed to prevent these events.

## Conclusion

Here, we describe a case of end-stage AtCM experiencing multiple cardiovascular events over three decades. We should be aware of AF/AT, bradyarrhythmia, CHF worsening, functional mitral and tricuspid regurgitation, and thromboembolism in patients with end-stage AtCM. To prevent these complications, comprehensive treatment strategies, including catheter ablation, oral anti-coagulation, CRT, and LAA closure, should be discussed.

## Data Availability

Data supporting the findings of this study are available from the corresponding author upon reasonable request.

## Lead author biography

Takafumi Oka is the EP chief doctor at Osaka University Hospital in Suita, Japan. His main areas of research interest are ablation therapy and CIEDs for patients with cardiomyopathy.
